# Efficacy of Long-Term Treatment of Autosomal Recessive Hypercholesterolemia With Lomitapide: A Subanalysis of the Pan-European Lomitapide Study

**DOI:** 10.3389/fgene.2022.937750

**Published:** 2022-08-22

**Authors:** Laura D’Erasmo, Antonina Giammanco, Patrizia Suppressa, Chiara Pavanello, Gabriella Iannuzzo, Alessia Di Costanzo, Daniele Tramontano, Ilenia Minicocci, Simone Bini, Anja Vogt, Kim Stewards, Jeanine Roeters Van Lennep, Stefano Bertolini, Marcello Arca, the Italian and European Working Group on Lomitapide in HoFH, Marcello Arca

**Affiliations:** ^1^ Department of Translational and Precision Medicine, Sapienza University of Rome, Rome, Italy; ^2^ Dipartimento di Promozione Della Salute Materno Infantile, Medicina Interna e Specialistica Di Eccellenza “G. D’Alessandro” (PROMISE), Università Degli Studidi Palermo, Palermo, Italy; ^3^ Department of Internal Medicine and Rare Diseases Centre “C. Frugoni”, University Hospital of Bari, Bari, Italy; ^4^ Centro E. Grossi Paoletti, Dipartimento di Scienze Farmacologiche e Biomolecolari, Università Degli Studi di Milano, Milan, Italy; ^5^ Department of Clinical Medicine and Surgery, Federico II University, Naples, Italy; ^6^ Medizinische Klinik und Poliklinik IV, Klinikum der Universität München, Munich, Germany; ^7^ Department of Internal Medicine, University Medical Centre Rotterdam, Rotterdam, Netherlands; ^8^ Department of Internal Medicine, University of Genova, Genova, Italy

**Keywords:** Real-world study, rare disease, autosomal recessive hypercholesterolaemia, LDL-C, lomitapide, long-term, efficacy, safety

## Abstract

**Background** **and aim:** Autosomal recessive hypercholesterolemia (ARH) is a rare autosomal recessive disorder of low-density lipoprotein (LDL) metabolism caused by pathogenic variants in the *LDLRAP1* gene. Like homozygous familial hypercholesterolemia, ARH is resistant to conventional LDL-lowering medications and causes a high risk of atherosclerotic cardiovascular diseases (ASCVDs) and aortic valve stenosis. Lomitapide is emerging as an efficacious therapy in classical HoFH, but few data are available for ARH.

**Results:** This is a subanalysis carried out on nine ARH patients included in the Pan-European Lomitapide Study. The age at starting lomitapide was 46 (interquartile range (IQR), 39.0–65.5) years, with a median treatment duration of 31.0 (IQR 14.0–40.5) months. At baseline, four (44.4%) patients had hypertension, one (11.1%) had diabetes mellitus, two (22.2%) were active smokers, and five (55.5%) reported ASCVD. The baseline LDL-C was 257.0 (IQR, 165.3–309.2) mg/dL. All patients were on statins plus ezetimibe, three were receiving Lipoprotein apheresis (LA), and one was also receiving proprotein convertase subtilisin/kexin type 9 inhibitors (PCSK9i). The addition of lomitapide (mean dose, 10 mg) resulted in the achievement of a median on-treatment LDL-C of 101.7 mg/dL (IQR, 71.3–138.3; 60.4% reduction from baseline), with a best LDL-C value of 68.0 mg/dL (IQR, 43.7–86.7; 73.5% reduction from baseline). During follow-up, one patient stopped both PCSK9i and LA. Recurrence of ASCVD events was reported in one patient. The median on-treatment aspartate transaminase and alanine transaminase values were 31.1 (IQR, 22.6–48.3) U/L and 31.1 (IQR, 27.2–53.8) U/L, respectively. Among six ARH patients with available fibroscan examination, liver stiffness values recorded at the last visit were within the normal range (median, 4.7 KPa; IQR, 3.6–5.3 KPa).

**Conclusion:** Lomitapide is effective and safe in ARH therapy as well as in classical HoFH.

## Introduction

Autosomal recessive hypercholesterolemia (ARH) is an ultrarare genetic disorder of lipid metabolism caused by disruptive variants in both alleles of the gene coding for the low-density lipoprotein receptor (LDLR) adaptor protein-1 (*LDLRAP1*) (D’Erasmo et al., 2020; Bertolini et al., 2020; Tromp et al., 2022; D’Erasmo et al., 2018). LDLRAP1 is a cytosolic protein required for the LDLR-mediated internalization of low-density lipoproteins (LDL) in polarized cells, such as hepatocytes (D’Erasmo et al., 2020; Bertolini et al., 2020; Tromp et al., 2022; D’Erasmo et al., 2018). This leads to a defective clearance of LDL particles from circulation, thereby causing a severe elevation of LDL cholesterol (LDL-C). ARH patients have an increased risk of premature atherosclerotic cardiovascular disease (ASCVD), which is comparable to that observed in homozygous familial hypercholesterolemia (HoFH) with defective pathogenic variants in the *LDLR* gene (D’Erasmo et al., 2020; Bertolini et al., 2020; Tromp et al., 2022; D’Erasmo et al., 2018). It is well known that in HoFH, the magnitude and duration of exposure to elevated values of LDL cholesterol (also called as LDL-C burden) largely determine the cardiovascular prognosis (D’Erasmo et al., 2020; Tromp et al., 2022; D’Erasmo et al., 2018). A recent survey on the natural history of ARH showed considerable heterogeneity in response to treatments but also highlighted that LDL-C in ARH patients stays far from the ideal target despite intensive treatment with conventional lipid-lowering therapies (including a combination of statins and/or ezetimibe and/or lipoprotein apheresis). As a consequence, their cardiovascular risk remains very high (D’Erasmo et al., 2020; D’Erasmo et al., 2018), thereby urging the identification of more effective treatments.

Monoclonal antibodies against proprotein convertase subtilisin/kexin type 9 inhibitors (PCSK9i) and lomitapide are the newly available therapies for severe forms of genetic hypercholesterolemia (D’Erasmo et al., 2021a). However, the efficacy of PCSK9i in ARH is currently disputed because the mechanism of their actions is largely dependent on the extent of residual LDLR functionality (D’Erasmo et al., 2021a; Cesaro et al., 2022). In contrast, lomitapide inhibits the activity of microsomal triglyceride transfer protein (MTP), which decreases the secretion of very-low-density lipoprotein (VLDL) by the liver, and, consequently, the production of LDL is derived from VLDL. Thus, lomitapide acts using an LDLR-independent mechanism, therefore potentially extending its efficacy in patients with an absent LDLR function, like those with ARH. Previous findings have suggested the existence of an inverse correlation between residual LDLR activity and MTP expression and activity, thereby probably causing an inverse correlation between LDLR activity and the effectiveness of a pharmacological MTP inhibition (Sirtori et al., 2014). Although preliminary observations have indicated the efficacy of lomitapide in ARH, the size of this benefit and, more importantly, its long-term safety has been poorly investigated. We have recently completed a real-world survey, including hypercholesterolemic patients with biallelic pathogenic variants in *LDLR* and *LDLRAP1* receiving lomitapide, in Europe (D’Erasmo et al., 2021b; D’Erasmo et al., 2021c). This unique cohort provides the opportunity to perform a subanalysis of the carriers of homozygous mutations in *LDLRAP1* to specifically evaluate the long-term LDL-lowering potential and safety of lomitapide in ARH.

## Material and Methods

### Study Design and Study Patients

This is a post hoc analysis of the Pan-European Lomitapide Study (D’Erasmo et al., 2021b) performed on a subgroup of patients diagnosed with ARH due to homozygous mutation in *LDLRAP1*. The Pan-European Lomitapide Study was a multicenter, observational, and retrospective survey collecting data from all patients known to be receiving lomitapide in Europe in the context of usual clinical practice and without protocol-mandated procedures (D’Erasmo et al., 2021b). The protocol of the Pan-European Lomitapide Study has been reported in detail elsewhere (D’Erasmo et al., 2021b). For a brief period, physicians were asked to retrospectively retrieve demographic and clinical information from medical records. Baseline data were defined as those at the date of initiation of lomitapide, whereas last follow-up data were those at the time of the last clinic visit up to December 31, 2019, respectively (D’Erasmo et al., 2021b). Details of concomitant lipid-lowering therapies, dosages of lomitapide, and side effects at each visit were requested to assess the efficacy and safety of this treatment (D’Erasmo et al., 2021b).

### Statistical Analysis

For descriptive statistics, continuous traits were presented as mean and standard deviation or as the median and interquartile range (IQR), as appropriate (D’Erasmo et al., 2021c). Categorical traits were shown as numbers and proportions. Comparisons were carried out using Mann–Whitney test for non-normally distributed variables and Student’s t-test for normally distributed variables (D’Erasmo et al., 2021c). For differences between categorical traits, the *p* value was calculated using the Chi-squared or Fisher’s exact test, as appropriate. Paired *t*-test was used to evaluate the difference between untreated, lowest, and last visit total and LDL-C, as well as LDL-C burden pre- vs. on-treatment. Linear regression with the enter method was used to evaluate associations (D’Erasmo et al., 2021c). Values that were not normally distributed were *log*-transformed before entering the model (D’Erasmo et al., 2021c).

Statistical analyses were performed using the IBM Statistical Package for Social Sciences (IBM SPSS, version 25.0, Inc. Chicago, IL, USA). A *p* value < 0.05 was considered statistically significant.

## Results

### Baseline Characteristics

The baseline characteristics of the nine ARH patients included in the Pan-European study are summarized in Table 1. Patients were equally distributed between sex, and they were middle-aged (median age, 52.1 years old; IQR, 43.9–67.4 years old). Three of the four women had been pregnant before the start of lomitapide. Most of them (*N* = 7) were from Italy and two were West Asian–European Turkish. Approximately 90% presented with xanthomata. They were slightly overweight, with a prevalence of current smoking (22.2%) and diabetes mellitus (11.1%). Moreover, approximately half of the patients had well-controlled hypertension. The majority (55.6%) had already experienced at least one ASCVD event before starting lomitapide and, among these patients, the mean age at the first event was 45.2 ± 7.9. Table 1 shows that the untreated mean LDL-C level was very similar to that observed in typical HoFH carrying defective *LDLR* pathogenic variants (Bertolini et al., 2020). Their median baseline LDL-C level was 257.0 mg/dL (IQR, 165.3–309.2) despite receiving a combination of lipid-lowering therapies that included statins and ezetimibe in all patients, evolocumab in one patient, and lipoprotein apheresis (LA) in three patients (one weekly, one biweekly, and one monthly). HDL-C and triglyceride levels were within normal ranges.

**TABLE 1:** Baseline characteristics of ARH patients

	Lomitapide cohort (*N* = 9)	
Demographic Variables		
Age, years (IQR)	52.1 (43.9–67.4)	
Male, *n* (%)	5 (55.6)	
Geographic Origin, *n* (%)		
European	7 (77.7)	
West Asian-European Turkish	2 (22.2)	
Xanthomas, *n* (%)	8 (88.9)	
ARH genotypes, *n* (%)		
c.430_431insA	5 (55.5)	
c.406C > T	1 (11.1)	
c.89–1 G > C	1 (11.1)	
Not provided	2 (22.2)	
Previous Pregnancy, *n* (%)	3 (42.9)	
Risk factors		
BMI, kg/m^2^ (IQR)	28.7 (22.2–35.9)	
Current Smoking, *n* (%)	2 (22.2)	
T2DM, *n* (%)	1 (11.1)	
Hypertension, *n* (%)	4 (44.4)	
PAS (mmHg)	120.0 (111.7–122)	
PAD (mmHg)	71.0 (67.7–80.0)	
Previous MACE, *n* (%)	5 (55.6)	
Age at first MACE, yrs	45.2 ± 7.9	
Plasma Lipids(mg/dl)	mg/dL	mmol/dL
Untreated		
Total cholesterol	569.5 (476.5–666.5)	14.72
LDL-C	464.0 (455.4–608.0)	11.99
Baseline		
Total cholesterol	337.0 (229.5–389.0)	8.71
LDL-C	257.0 (165.3–309.2)	6.64
HDL-C	51.0 (43.0–57.5)	1.32
Total triglycerides	123.0 (81.8–153.5)	1.39
Lipid lowering therapies, *n* (%)		
None	0	
Statin	9 (100.0)	
Ezetimibe	9 (100.0)	
PCKS9i	1 (11.1)	
Fibrates	0	
LA	3 (33.3)	
weekly	1 (11.1)	
bi-weekly	1 (11.1)	
Monthly	1 (11.1)	

Data are represented median (interquartile range) and number (percentage) as appropriate.

The worst lipid profile without any cholesterol lowering medication is reported as naïve values. Percentage associated with genotypes are reported on the whole cohort.

ARH, autosomal recessive hypercholesterolemia; LDL-C, low density lipoprotein cholesterol; HDL-C, high density lipoprotein cholesterol; BMI, body mass index; T2DM, type 2 diabetes; LA, lipoprotein apheresis; PCKS9i, Proprotein convertase subtilisin/kexin type 9 inhibitors; MACE, major atherosclerotic cardiovascular events.

### Efficacy Analysis

**FIGURE 1:**
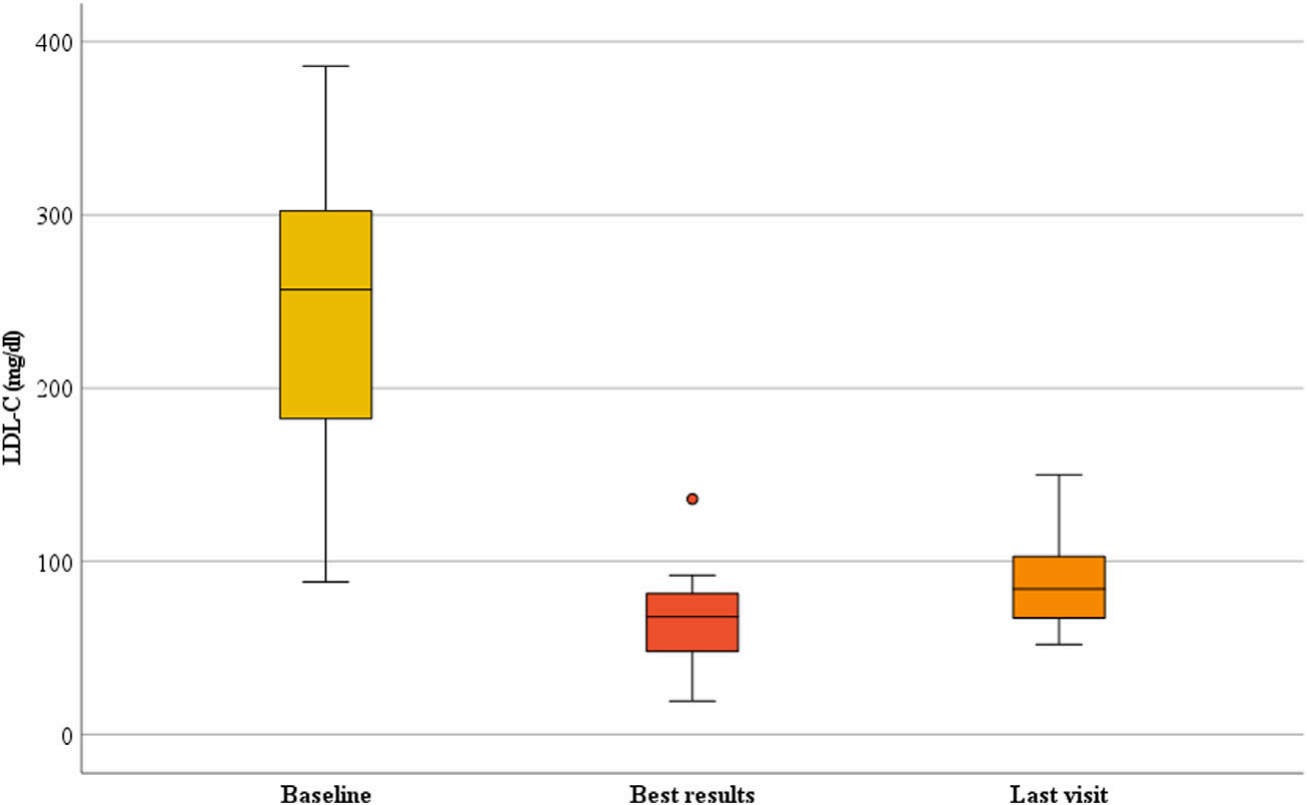
Change in LDL-C during lomitapide therapy in ARH patients. Box plot graphs represent the median LDL-C levels in the ARH population receiving lomitapide. The yellow, red, and orange boxes represent the median LDL-C, nadir on-treatment, and last visit results, respectively. LDL-C, low-density lipoprotein cholesterol.

**FIGURE 2:**
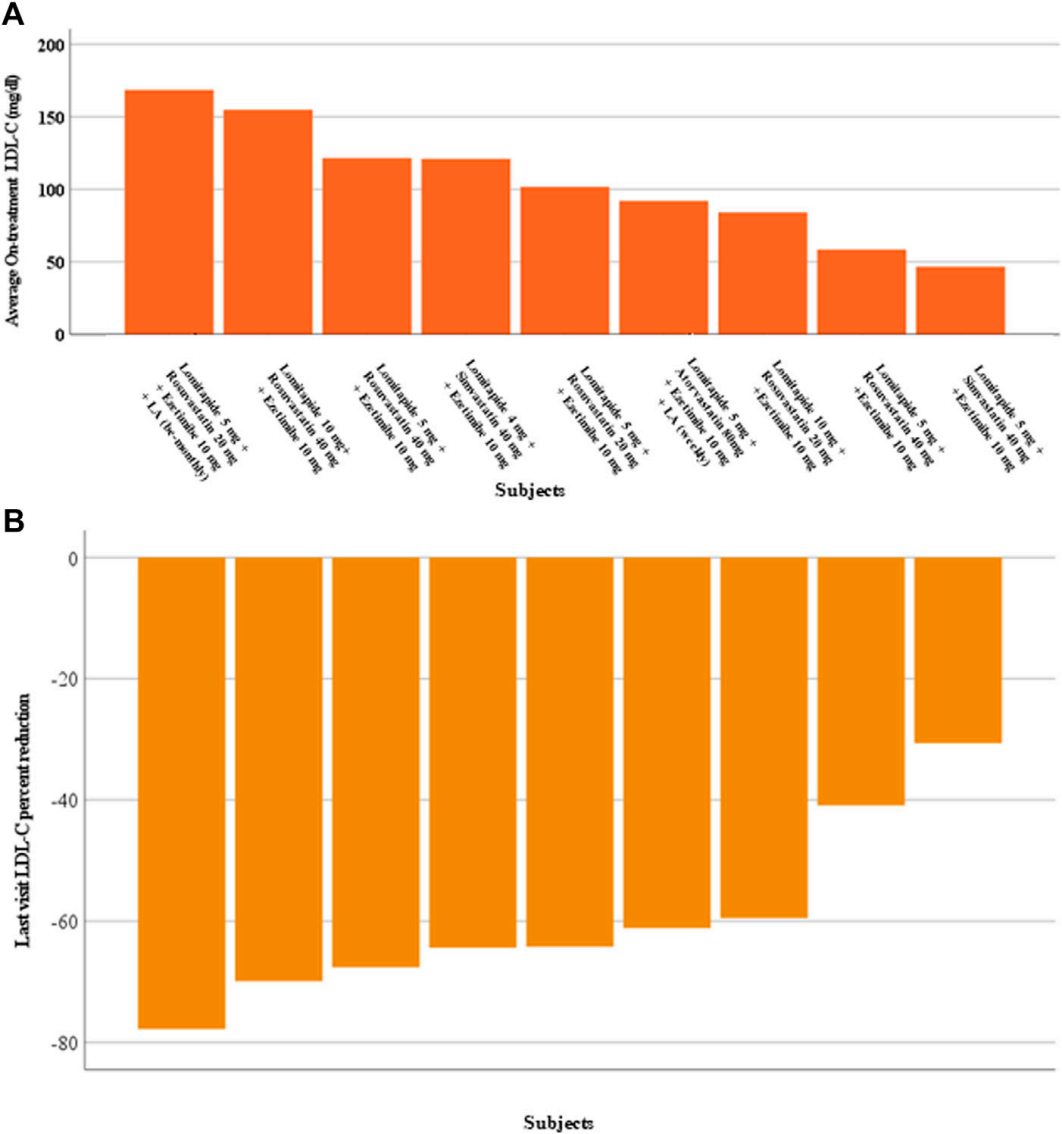
Individual changes in LDL-C levels in ARH patients during lomitapide therapy. **(A)** This figure represents the individual achieved average on-treatment LDL-C. The therapies prescribed to the patients in the last visit are reported in the *x*-axis. **(B)** This figure represents the individual percent reduction in LDL-C from the baseline to the last visit. LDL-C, low-density lipoprotein cholesterol.

The median duration of follow-up of lomitapide treatment was 31 months (IQR, 14.0–50.5; range, 13–59 months). The addition of a mean dose of 10 ± 11.4 (IQR, 5–10) mg/day of lomitapide allowed the achievement of the best median on-treatment LDL-C value of 68.0 mg/dL (IQR, 43.7–86.7; *p* < 0.001) corresponding to a percent reduction from baseline of 72.0% (IQR, 59.7–79.2; Figure 1). At the last visit, a slight rise in the median value of LDL-C to 84.0 mg/dL (IQR, 66.1–115.4) was observed, thereby bringing the percentage reduction of LDL-C at the end of the follow-up to 64.2% from the baseline (IQR, 50.2–68.7; *p* < 0.001; Figure 1). Overall, the median on-treatment LDL-C was 101.7 mg/dL (IQR, 71.3–138.3), with wide intraindividual variability, ranging from a minimum to of 46.7 mg/dL to a maximum of 168.5 mg/dL (Figure 2A). When percentage change was considered from the baseline (Figure 2B), ARH patients displayed individual reductions ranging from a minimum of 30.6% to a maximum of 77.8%, but differences in the effects were independent of the lomitapide dose at the last visit (*P* = NS). Comparable results were obtained if the absolute LDL-C reduction was considered (data not shown). Notably, this lipid-lowering effect was obtained despite six patients receiving only 5 mg/day of lomitapide (Figure 2A). In the ARH patient receiving the quadruple combination of atorvastatin 40 mg, ezetimibe 10 mg, monthly LA, and evolocumab (baseline LDL-C of 461 mg/dL), the addition of lomitapide allowed the interruption of evolocumab and LA after 3 months due to a drop of 83% in LDL-C from baseline (Figure 2).

To identify possible explanations for variability in the individual lipid response, we performed univariate and multivariate regression analysis to show that the achieved on-treatment LDL-C levels were independent of the duration of follow-up, sex, age at lomitapide start, untreated LDL-C, background therapy, or lomitapide dosage (data not shown).

### Safety Analysis

Data on gastrointestinal side effects were not available for all patients at each timepoints. However, the most common side effect was diarrhea followed by nausea, which was reported in two patients. One patient complained of diarrhea with a mild-to-moderate intensity at each visit and she was prescribed nutritional counseling from the referring physician with benefit. She had to stop treatment because she had moved to another country, where lomitapide is not allowed. Another patient referred to the same gastrointestinal side effect and was prescribed with diet; however, he showed very poor adherence to the dietary regimen and lomitapide treatment. Indeed, he stopped lomitapide after 18 months of treatment due to gastrointestinal side effects and lack of compliance.

**FIGURE 3:**
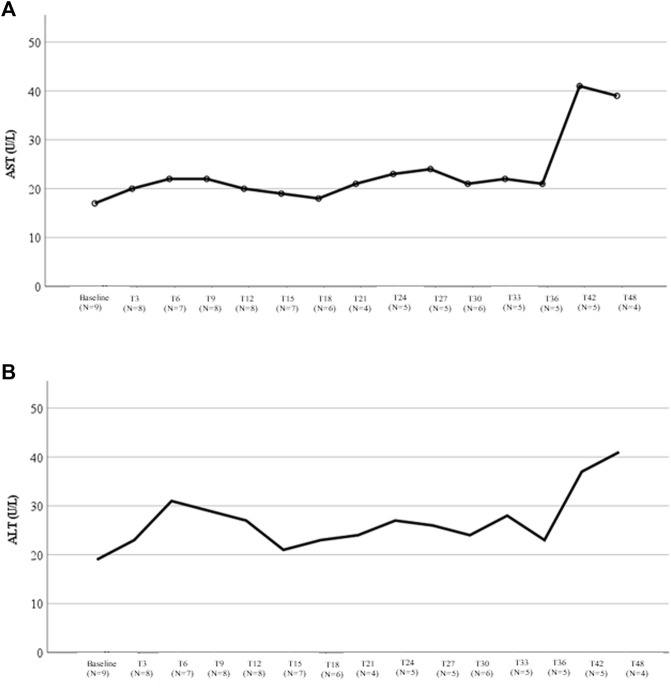
Liver transaminases variations during therapy with lomitapide in ARH patients. **(A)** This figure represents the variation in the median AST value during treatment with lomitapide according to the months of treatment. For each timepoint, the number of patients with available data was reported. **(B)** This figure represents the variation in the median ALT value during treatment with lomitapide according to months of treatment. For each timepoint, the number of patients with available data was reported. AST, aspartate aminotransferase; ALT, alanine aminotransferase.

Changes in liver function tests during lomitapide treatment are shown in Figure 3. Aspartate aminotransferase (AST) levels always remained <40 U/L (Figure 3A) and the median on-treatment value was 31.1 U/L (IQR, 22.6–48.3 U/L). Among the four ARH patients with the longest follow-up (4 years), the median AST was 22 U/L (IQR, 16.2–36.0; range, 16–39 U/L). A similar trend was observed for alanine aminotransferase (ALT) values, whose median on-treatment value was 31.1 U/L (IQR, 27.2–53.8; Figure 3B). In patients with a longer follow-up, the median ALT value was 17.5 U/L (IQR, 13.5–35.7 U/L; range, 13–41 U/L).

Liver ultrasound was available in seven and eight patients at baseline and last visit, respectively (Table 2). Two ARH patients did not show changes in the severity of liver fat, while four experienced worsening of the hepatic fat content. Even though only one patient reported a fibroscan evaluation at baseline, this information was available in six ARH patients at the last visit. In this subgroup of patients exposed to lomitapide for a median period of 35 months (IQR, 14.0–40.2), the individual values of hepatic stiffness remained well within the normal range of <7.0 KPa (median, 4.6 KPa; range, 3.5–6.3 kPa).

**TABLE 2:** Baseline and last visit measurements of hepatic steatosis and stiffness in individual ARH patients treated with lomitapide

Subjects	Baseline		Last Visit	
	Ultrasound	Fibroscan	Ultrasound	Fibroscan
1	—	—	moderate	4.5
2	—	—	moderate	3.5
3	absent	—	moderate	4.8
4	Mild	—	mild	
5	absent	—	mild	3.6
6	moderate	—	Moderate	4.9
7	absent	5.7	Moderate	6.3
8	absent	—	—	—
9	absent	—	Moderate	—

Data are represented as value per each subject by reporting ultrasound and fibroscan data as previously described (D’Erasmo et al., 2021b).

For comparison data are reported at baseline and last visit. The severity of hepatic steatosis was estimated by an ultrasonographical semi-quantitative measurement of liver fat content and categorized as absent, mild, moderate, or severe (D’Erasmo et al., 2021b).

In the entire group, only one patient experienced an elevation in liver function test at approximately thrice the upper limits of the normal after 15 months of treatment. AST/ALT levels returned to normal range at the next laboratory check without any change in lomitapide dosage. This patient also experienced a change in hepatic fat, from absent to moderate steatosis, at the last follow-up visit. However, the patient stopped lomitapide a few months after the liver function test elevation due to gastrointestinal side effects and poor treatment compliance.

### ASCVD Outcomes

Six patients had internal carotid plaques at baseline ultrasound examination and none had progression during follow-up. Data on common carotid intima media thickness (CCA-IMT) were available in four of nine patients showing no progression of subclinical atherosclerosis. In this subgroup, the median CCA-IMT was 1.4 (0.9–2.4) mm on the right and 1.4 (1.0–1.6) mm on the left at the first lomitapide prescription. After more than 2 years of treatment, the median CCA-IMT was 1.4 (1.0–2.4) mm on the right and 1.2 (0.7–1.7) mm on the left (paired *t*-test, *P* = NS). One patient experienced a recurrence of ASCVD events during follow-up due to the worsening size of the abdominal aorta aneurism, coronary revascularization, and one episode of acute heart failure.

## Discussion

The results from the present analysis confirmed over approximately 3 years of treatment the efficacy and the reassuring safety profile of lomitapide in ARH patients. Indeed, the adjunct of lomitapide to background lipid-lowering therapies allowed up to a 70% reduction in LDL-C from baseline, thereby resulting in an on-treatment LDL-C of 101.7 mg/dL. Moreover, neither liver function tests nor indicators of liver fibrosis showed appreciable changes in these patients during the exposure to lomitapide.

ARH patients enrolled in the present survey showed some differences compared with those previously reported by D’Erasmo *et al.* (2018). To this regard, it is interesting to note that in the Pan-European cohort (D’Erasmo et al., 2021b), we observed that ARH showed higher body mass index (BMI) compared to null/null homozygous FH patients (28.9 ± 6.6 units vs. 23.6 ± 4.2 units, respectively; *p* = 0.047). Interestingly, this difference was independent of age and gender. A recently published paper by Leigh *et al.* (2022) has demonstrated that *LDLRAP1* ^*−/−*^ mice on a chow diet gained significantly more weight and were more insulin resistant compared with control mice. Further studies are needed to confirm this observation in humans carrying bi-allelic mutations in the *LDLRAP*1 gene.

The present findings confirm those previously reported on the efficacy of lomitapide in six patients included in the paper by D’Erasmo et al. (2018), thereby showing that lomitapide reduced LDL-C from baseline up to approximately 80% with a mean dosage of approximately 20 mg/day. In the present study, longer data on the efficacy and safety of the use of lomitapide in ARH patients compared with the previously published data were included, thereby adding more granular information on the hepatic safety that was lacking in the previous observation. In addition, all the patients in the previous paper were from Italy. However, in this collection, we also retrieved information on two ARH patients treated outside Italy: one from Germany and another from the Netherlands. It must be noted that the average dosage of 10 mg/day of lomitapide recorded in the present survey was different from that of 20 mg/day reported in a previous study (D’Erasmo et al., 2018). This discrepancy could be explained, at least partially, with the fact that the two group of ARH patients were different. In fact, only four participated in the two surveys and two of these were enrolled in the phase 3 trial, where an escalating protocol from 5 to 60 mg/day was scheduled.

According to what observed in HoFH (D’Erasmo et al., 2021b; D’Erasmo et al., 2021c), the present study showed wide variability in the lipid-lowering response, which is difficult to explain. In the Pan-European cohort (D’Erasmo et al., 2021b), changes in LDL-C levels were obtained with a mean lomitapide dose at the last visit of 21.2 ± 14.4 mg/day in the HoFH defective/defective group, 6.7 ± 2.9 mg/day in the null/defective group, 19.1 ± 9.8 mg/day in the null/null and 10 ± 11.4 mg/day in the ARH group (data not shown). Therefore, as already shown (D’Erasmo et al., 2021b), the LDL-C lowering effect of lomitapide was independent of the residual LDLR activity and heterogeneity in the results may be explained by other factors. We can only speculate that ancestry (null/null and null/defective are mainly from abroad Italy, whereas most defective/defective and ARH are Italian), dietary habits (Perry, 2013), adherence to medications, polymorphism in the *MTP* gene (Ledmyr et al., 2002), gut microbiota characteristics, and bile metabolism (Patel et al., 2022) influence the lipid-lowering effect of lomitapide. Unfortunately, these factors were not investigated in the present work, which prevented the making of any further consideration.

Even if a direct comparison is not feasible and is out of the scope of the present study, discussing the differences in the response to PCKS9 inhibitors and lomitapide in ARH is important. The TESLA part B trial, in which 49 patients were treated with evolocumab 420 mg or placebo Q4W for 4 weeks on top of the ongoing lipid lowering therapy, reported a 30.9% reduction in LDL-C compared to baseline (Raal et al., 2015). When analyzed according to *LDLR* pathogenic variants status, the patients with *LDLR* null variants in both alleles or those with autosomal recessive HoFH did not respond to evolocumab treatment (Raal et al., 2015). A heterogeneous reduction in LDL-C levels’ response to PCSK9i was also experienced in the treatment of ARH in Spain: three patients received triple and combined treatment with PCSK9i (evolocumab) as well as obtained heterogeneous responses ranging from 19% to 59% reduction in LDL-C (Sánchez-Hernández et al., 2018). In contrast, with the adjunct of lomitapide, a median percent reduction of 64.2% (IQR, 50.2–68.7) at the last visit was observed with the lowest percentage of LDL-C reduction value of approximately 30%. Although both PCSK9i and lomitapide are associated with heterogeneity in the LDL-C lowering efficacy and a face-to-face comparison has never been done so far, the data of the current study suggest that discussing the possibility to tailor lipid-lowering treatments to a patient’s genetic background within the scientific community can be timely, thereby potentially improving the cost–benefit balance. In the case of ARH, treatment based on the LDLR activity will not allow the achievement of LDL-C targets and, therefore, this may not be prescribed.

The analysis of the safety outcomes highlights that lomitapide is a manageable drug in ARH. According to other studies (D’Erasmo et al., 2021a; D’Erasmo et al., 2021b), gastrointestinal side effects were managed with a dietary regimen prescription. Only one patient stopped lomitapide due to diarrhea, but the responsible physicians referred poor adherence to a low-fat diet. Nutritional support may be useful for handling patients treated with lomitapide because gastrointestinal side effects in most cases are preventable and manageable with a dietary plan (D’Erasmo et al., 2021a; D’Erasmo et al., 2021b).

Even though an overall increase in liver fat, as estimated by ultrasounds, was observed in most patients, the hepatic steatosis was always described as moderate and never severe at the last follow-up visits. In the group of ARH patients who received fibroscan evaluation during follow-up, liver stiffness remained within the normal range, thereby suggesting that the increase in fat liver content was not necessarily associated with fibrosis. Moreover, the results observed in the present ARH cohort were obtained despite most patients receiving only 5 mg/day of lomitapide at the last visit. The low dose of lomitapide (mean dose, 10 mg/day) used in this population could have minimized the risk of long-term liver-associated side effects. However, the small sample size and the relatively short period of observation prevent giving any definite conclusion.

An interesting aspect to be considered is whether lomitapide may cause different liver side effects in ARH vs. HoFH. The present report was not designed to answer this question, and the findings of the Pan-European study should be referred to (D’Erasmo et al., 2018). Although no difference between HoFH and ARH in the occurrence of liver function test elevation after lomitapide was detected, an increase in liver steatosis was particularly evident among ARH patients (data not shown). Thus, ARH patients had a 2.6 (95% CI, 1.0–6.8) higher risk of having moderate steatosis compared with other genotypes, independent of age, gender, and lomitapide dose. The dysregulation in lipid storage and energy homeostasis highlighted in mice in the study by Leight *et al.* (2022) could explain this observation. However, the present data prevent us from making any definite conclusion. Moreover, whether the increase in hepatic fat content in ARH may translate into liver damage is unknown. However, the lack of change in liver stiffness up to 3 years of follow-up report in the present study argues against the signal of liver fibrosis in ARH patients exposed to lomitapide.

As an exploratory analysis, the occurrence of ASCVD during lomitapide exposure was evaluated. No progression of carotid artery disease was observed. Only one of the nine patients suffered from ASCVD recurrence. It must be noted that this patient was at really high risk as she was in her sixties and already had cardiovascular disease at the time of initiation of lomitapide. In addition, she had other cardiovascular risk factors beyond LDL-C elevation as smoking habits, hypertension, and type 2 diabetes. Although anectodical, we believe that these observations are suggestive of a potential benefit of lomitapide in halting the progression of atherosclerotic damage. Thus, additional studies focused on assessing the cardiovascular benefit of lomitapide in ARH/HoFH patients are needed to give a definite answer to this question.

## Limitations

This study has several limitations that must be acknowledged. The retrospective nature of the study is the most important. Moreover, patients did not receive follow-up according to a prespecified protocol, and the management of each patient was entirely based upon the judgment of the treating physicians. This analysis included only a relatively small cohort of ARH patients. Therefore, the expansion of similar analyses to larger cohorts of ARH patients is strongly recommended. However, it should be considered that ARH is an ultrarare disease and the largest worldwide cohort described concerned 52 patients (D’Erasmo et al., 2018).

## Conclusion

Treating ARH is an unmet clinical need because these patients are far from the recommended LDL-C goals with the conventional lipid-lowering therapies. The findings of the current study strongly indicate that lomitapide should be considered an effective and safe LDL-lowering therapy for ARH patients, which is similar to that reported in classical HoFH (Wilemon et al., 2020).

## The Members of the Italian and European Working Group on Lomitapide in HoFH

Marcello Arca, Simone Bini, Alessia Di Costanzo, Laura D’Erasmo (Department of Translational and Precision Medicine, Sapienza University of Rome); Maurizio Averna, Angelo Baldassare Cefalù, Antonina Giammanco (Dipartimento di Promozione Della Salute Materno Infantile, Medicina Interna e Specialistica Di Eccellenza “G. D’Alessandro” (PROMISE), Università Degli Studi di Palermo); Livia Pisciotta, Stefano Bertolini (Department of Internal Medicine, University of Genova); Eric Boersma (Department of Cardiology, University Medical Center Rotterdam); Katia Bonomo, Fabio Nota (Metabolic Disease and Diabetes Unit, AOU San Luigi Gonzaga, Orbassano, Torino); Marco Bucci (Department of Medicine and Aging Sciences, University “G. D'Annunzio” of Chieti-Pescara); Laura Calabresi, Chiara Pavanello (Centro E. Grossi Paoletti, Dipartimento di Scienze Farmacologiche e Biomolecolari, Università Degli Studi di Milano, Milano); Paolo Calabrò, Arturo Cesaro (Department of Translational Medical Sciences, University of Campania “Luigi Vanvitelli”, Division of Cardiology, A.O.R.N. “Sant’Anna e San Sebastiano”, Edificio C - Cardiologia Universitaria, Caserta 81100); Jaimini Cegla (Lipid and Cardiovascular Risk Service, Imperial College Healthcare NHS Trust, London); Sergio D'Addato, Fulvio Ventura (Hypertension and Cardiovascular Risk Factors Research Center, Medical and Surgical Sciences Department, Sant'Orsola-Malpighi University Hospital); Eugene Daphnis (Nephrology Department, University Hospital of Heraklion, Crete); Maria Donata Di Taranto, Giuliana Fortunato (Department of Molecular Medicine and Medical Biotechnology, CEINGE-Biotecnologie Avanzate, University of Naples Federico II); Avishay Ellis (Internal Medicine, Rabin Medical Center, Petah Tikva); Fabio Fimiani (Unit of Inherited and Rare Cardiovascular Diseases, A.O.R.N. Dei Colli “V. Monaldi”, Naples); Marco Gentile, Gabriella Iannuzzo (Department of Clinical Medicine and Surgery, Federico II University, Naples); Meral Kayikcioglu (Department of Cardiology, Ege University School of Medicine, Izmir), Genovefa Kolovou (Cardiometabolic Center, Lipoprotein Apheresis and Lipid Disorders Clinic, Metropolitan Hospital, Athens); Evangelos Liberopoulos (Department of Endocrinology, Police Medical Centre of Thessaloniki, Greece and Department of Internal Medicine, Medical School, University of Ioannina, Ioannina); Karin Littmann (Unit of Endocrinology, Department of Medicine Huddinge, Karolinska Institutet, Theme Inflammation and Ageing, Karolinska University Hospital, Stockholm); Sergio Martínez-Hervás (Service of Endocrinology and Nutrition, Hospital Clínico Universitario of Valencia, Spain and INCLIVA Biomedical Research Institute, CIBER de Diabetes y Enfermedades Metabólicas Asociadas (CIBERDEM), ISCIII, Spain and Department of Medicine, University of Valencia, Valencia); Tiziana Montalcini (Department of Clinical and Experimental Medicine, University “Magna Græcia” of Catanzaro, Catanzaro); Arturo Puja (Department of Medical and Surgical Sciences, University “Magna Græcia” of Catanzaro, Catanzaro); José Real (Institute of Health Research-INCLIVA, Spain and Endocrinology and Nutrition Service, University Clinic Hospital of Valencia, Valencia and CIBERDEM, Diabetes and Associated Metabolic Diseases Networking Biomedical Research- ISCIII, Madrid); Joost Rutten, Kim Steward, Jeanine Roeters van Lennep, (Department of Internal Medicine, University Medical Centre Rotterdam); Carlo Sabbà, Patrizia Suppressa (Department of Internal Medicine and Rare Diseases Centre “C. Frugoni”, University Hospital of Bari, Bari); Tiziana Sampietro, Francesco Sbrana (Lipoapheresis Unit-Reference Center for Diagnosis and Treatment of Inherited Dyslipidemias, Fondazione Toscana “Gabriele Monasterio”, Pisa); Anja Vogt (Medizinische Klinik und Poliklinik IV, Klinikum der Universität München, Munich); Giovanni Battista Vigna (Medical Department, Azienda Ospedaliero-Universitaria di Ferrara, Ferrara); Shahenaz Walji(Hammersmith Hospital Lipid Clinic, Imperial College Healthcare NHS Trust, London)

## Data Availability

The raw data of this work will be shared upon reasonable request by the corresponding author. Requests to access the datasets should be directed to Laura D'Erasmo, laura.derasmo@uniroma1.it.
